# A single Center study of the Symbol Digit Modalities test as a screening tool for cognitive impairment in Parkinson’s disease

**DOI:** 10.1016/j.prdoa.2025.100395

**Published:** 2025-09-16

**Authors:** Daniyell Thomason, Morganne Manuel, Shannin Moody, Jesus Lovera, Brain Copeland, Deidre Devier

**Affiliations:** a Louisiana State University Health Sciences Center – New Orleans, United States; b Medical College of Georgia – Augusta University, United States

**Keywords:** Cognition, Cognitive impairment, Parkinson’s disease, Neurodegeneration

## Abstract

**Background:**

Parkinson’s Disease (PD) can include physical signs and possibly cognitive impairment, resulting from the convergence of pathological processes involving dopaminergic dysfunction, accumulation of alpha-synuclein, cholinergic deficits, and disruption of other neurotransmitter systems. We used screening tests to evaluate the characteristics of cognitive performance in patients with PD and to assess their validity compared to the Montreal Cognitive Assessment (MoCA).

**Methods:**

This is a natural history study of participants with PD and controls screened for possible cognitive impairment using the MoCA, Symbol Digit Modalities Test (SDMT), and King-Devick (KD). The groups were compared on performance and then factors associated with cognitive performance (age, diagnosis, and level of education) were analyzed to determine which best predicted test scores.

**Results:**

SDMT scores were lower in the PD group (Mean = 36.7 ± 12.4) compared to controls (Mean = 47.2 ± 11.0, p < 0.001), but the MoCA (PD = 23.8 ± 3.5; Control = 25.5 ± 3.6, p = 0.02) and KD (PD = 70.1 ± 23.4 s; Control = 61.6 ± 17.5, p = 0.048) did not differentiate between groups after controlling for multiple comparisons. Age and diagnosis predicted SDMT raw scores and, as expected, only diagnosis remained significant after calculating T-scores based on published test norms. Age, education, and diagnosis predicted MoCA scores.

**Conclusions:**

The SDMT emerged as a promising screening tool to detect cognitive impairment in PD. The test’s age and education corrected norms controlled for those variables and left diagnosis as the only predictor of performance. The MoCA scores were predicted by age, education, and diagnosis suggesting the education correction of the MoCA did not fully account for the influence of demographic variables.

## 1 Background

Parkinson’s Disease (PD), the second most prevalent neurodegenerative disease, results from the loss of dopaminergic neurons in the substantia nigra pars compacta of the midbrain and the development of Lewy bodies [1,2] and is typically associated with tremors, bradykinesia, stiffness, rigidity, and cognitive impairment [3]. Cognitive dysfunction can manifest prior to diagnosis, at the time of diagnosis, or later, and the severity and rate of progression exhibit significant variability. Multiple cognitive domains are affected in those with PD, including attention, memory, visuospatial abilities and especially executive functions (mental flexibility, set shifting, switching, efficiently planning future actions and solving problems) [2].

Mild cognitive impairment (MCI) is significantly more common in people with PD than in the general population [4]. The Montreal Cognitive Assessment (MoCA) is a valid and reliable predictor of MCI due to its high sensitivity to subtle cognitive deficits [5] with utility in screening those with PD [6]. While the MoCA has been established as an effective tool for detecting MCI, more recent research indicates that it may overestimate cognitive impairment in older adults by failing to adequately account for the influence of age and education [7].

Another notable screening test sensitive to detecting not only the presence of brain damage but also subtle changes in cognitive functioning over time is the Symbol Digit Modalities Test (SDMT) [8]. The SDMT measures cognitive processing speed, attention, and visual scanning. Performance on the SDMT is a crucial predictor of conversion from normal cognition to MCI. Its brevity and ease of administration, normative data accounting for the age and education, along with its unambiguous scoring, make the SDMT a test of choice [9]. Little work has been completed to determine if the SDMT is useful to screen for cognitive impairment in patients with PD, though Pascoe, et. al. found it differentiated between participants with PD without MCI and those with MCI [9].

The King Devick Test (KD) test is a rapid number naming test that serves as a proficient and reliable tool for assessing visual tracking and saccadic eye movements. Initially created for the evaluation of reading proficiency, its application of has expanded to encompass the identification of diverse neurological conditions including PD [10] The primary function of the K-D test is to detect subtle impairment in eye movements and identify potential deficits in brain function associated with neurological conditions [11,12].

### 1.1 Objective

We used the SDMT and KD to evaluate their utility in screening for cognitive impairment in patients with PD and to assess their validity compared to the MOCA.

## 2 Methods

### 2.1 Participants

This is a natural history study of a convenience sample of participants with PD and controls. Adults diagnosed with PD using the UK Brain Bank Criteria and controls were selected from a larger cohort of individuals participating in a study designed to validate cognitive screening tools in people diagnosed with neurodegenerative disorders [13]. They were recruited from a multispecialty clinic neurology practice located in the Gulf South. Study team members identified patients who were eligible for study enrollment and medical records were reviewed to confirm inclusion and exclusion criteria were met. All study-related procedures occurred after completing the informed consent process. This study was approved by the institutional review board.

Demographic information included age (in years) and level of education (continuous variable based on last full year completed), self-reported sex (binary) and race (American Indian / Alaskan Native, Asian, Native Hawaiian or Pacific Islander, Black or African American, White), and ethnicity (Hispanic or Non-Hispanic). Years of education after high school were only counted if participants continued in a 4-year institution; this meant that trade schools and community colleges were not counted towards additional years of education. Disease duration was determined by measuring the time from diagnosis to the day of the study visit.

### 2.2 Inclusion criteria PD cohort

Adult diagnosed with Parkinson’s Disease.

### 2.3 Exclusion criteria both cohorts

Neurological disease including stroke, epilepsy, or other neurodegenerative disorders not included in our target list; an acute, severe or unstable medical condition; unable to speak English fluently.

### 2.4 Cognition

Cognitive screening used the MoCA, SDMT, and KD. The MoCA, a cognitive screening tool used to assess for MCI and validated for use with PD [5] was administered using the standardized methods delineated by the test guidelines or adapted for use in virtual visits due to the COVID 19 pandemic [14]. The score was totaled from all correct answers and 1 point was added for participants with 12 or fewer years of education. The highest possible score was 30. The MoCA items can be grouped to provide domain-specific index scores evaluating the following: memory, visuospatial/ Executive Function, attention, naming, abstraction, language, and orientation [15].

The SDMT assesses cognitive processing speed and consists of a key that matches symbols to numbers. The oral version was chosen to reduce confounding upper extremity motor impairment commonly seen in PD, but there could also be speech difficulties in some participants. The test involved 90 s of the participant quickly stating aloud the number that went with each symbol in succession. The tester recorded answers with the score including the total number of correct responses. Raw scores were converted into T-scores accounting for age (18–24, 25–34, 35–44, 45–54, 55–64, 65–78) and education (≤12 years or > 12 years).

The KD involved participants quickly reading aloud a series of numbers presented on cards or on a screen and measuring the time it took to complete the task accurately. Raw scores were converted to T-scores based on norms accounting for age (15, 16–19, 20–24, 25–29, 30–34, 35–39, 40–44, 45–49, 50–54, 55–59, 60–64, 65–69, 70–74, 75–79, 80–84, 85–89, 90–94).

### 2.5 Statistical analysis

All statistical analyses were completed using SPSS version 26. The demographic characteristics of the cohort were characterized using descriptive analysis. The control group was approximately matched to the PD group based on age and years of education. T-scores were calculated based on available normative data for both the SDMT published test manual [16] and KD (KD Pro Monitoring software). Two-way ANOVAs were employed to compare the cohorts (PD and control) on demographic variables and performance on the MoCA, SDMT, and K-D tests, and the number of participants that fell into the impaired range for each test. Linear Regression analyses were completed to assess the effect of variables (age, diagnosis, and education) on cognitive outcomes using stepwise removal for non-significant variables. Separate regression analyses were completed on the PD cohort to assess the relationship between disease duration and cognitive screening test scores. Disease duration did not predict test scores (data not shown).

Subsequently, Pearson's correlation analyses were performed to assess the relationship between the subcategories of the MoCA and SDMT scores. As this study was comprised of a convenience sample, no power analysis was performed.

## 3 Results

The PD cohort (n = 40), average age 66.8 years, included 14 females/26 males, 2 Black/38 White, and 1 Hispanic/39 non-Hispanic participants [17]. The average level of education was 14.9 years (standard deviation SD 2.4). The control cohort (n = 54), average age 65.6 (SD 8.1), included 33 females/21 males, 11 Black/42 White, and 2 Hispanic/52 non-Hispanic participants. The average level of education was 14.2 years (SD 3.0). The groups did not differ on age or education, two influential factors for cognitive performance. (Table 1).

**Table 1:** Demographics.

	PD (n = 40)	Control (n = 54)	F	p
Age (M, SD)	66.8, 9.1	65.6, 8.1	0.44	0.51
Education (M, SD)	14.9, 2.4	14.2, 3.0	1.52	0.22
Sex (%)	35 % female 65 % male	61 % female 39 % male		
Race	5 % Black 95 % White	21 % Black 79 % White		
Ethnicity	3 % Hispanic 97 % Non-Hispanic	4 % Hispanic 96 % Non-Hispanic		

Comparing the cohorts on cognitive performance, the PD group performed worse than the control group on the SDMT raw scores (F = 19.06, ηp^2^ = 0.17, 95 % CI [40.2, 45.4]) and SDMT T-scores (F = 17.94, ηp^2^ = 0.16, 95 % CI [40.4, 45.0]), but not on the MoCA (F = 5.63, ηp2 = 0.0.06, 95 % CI [24.0, 25.5), KD raw scores (F = 4.02, ηp2 = 0.04, 95 % CI [61.0, 69.4]) or KD T-scores (F = 3.56, p = 0.04, 95 % CI [38.2, 42.8]) after Bonferroni correction for multiple comparisons (p ≤ 0.01). (Table 2) Participants were considered below cutoff for ‘impaired’ if they scored < 26 on the MoCA or had a T-score ≤ 40 on the SDMT or KD. For the SDMT, 60 % of PD and 22 % of controls were impaired. For the MoCA, 73 % of PD and 39 % of controls fell in the impaired range and the KD classified 50 % of PD and 41 % of controls as impaired.

**Table 2:** ANOVA comparison of PD and control groups.

	PD (n = 40)	Control (n = 54)	F	ηp^2^			95 %CI
SDMT (M, SD)	36.7, 12.4	47.2, 11.0	19.06	0.17			40.2, 45.4
SDMT T-score (M, SD)	37.5, 10.3	46.5, 10.1	17.94	0.16			40.4, 45.0
MoCA (M, SD)	23.8, 3.5	25.5, 3.6	5.63	0.06			24.0, 25.5
KD (M, SD)	70.1, 23.4	61.6, 17.5	4.02	0.04			61.0, 69.4
KD T-score (M, SD)	38.0, 11.0	42.3, 11.1	3.56	0.04			38.2, 42.8

M = mean, SD = standard deviation

Further regression analyses focused on factors (age, diagnosis, and level of education) that predicted scores on the SDMT and MoCA. For the SDMT raw scores, age (β = -0.63, CI [-0.89, −0.38]) and diagnosis (β = -10.13, CI [-14.45, −5.81]) were significant factors (Table 3). However, only diagnosis (PD or control; β = -8.44, CI [-12.63, −4.25]) remained significant in the model including the T-scores based on the SDMT norms that account for age and education suggesting the T-score conversion adequately controlled for demographic factors (Table 3). Age (β = -0.12, CI [-0.20, −0.03]), education (β = 0.31, [1.16, 0.37]), and diagnosis (β = -1.85, CI [-3.27, −0.43]) were significant predictors of MoCA scores (Table 3). Thus, the 1-point score correction for years of education on the MoCA was an insufficient control in this cohort. In the PD group, disease duration was not a significant factor in predicting MoCA or SDMT scores. No further results are reported on the KD as it did not discriminate between groups and no demographic factors or diagnosis were predictors of scores. While the MoCA did not survive correction for multiple comparisons as a discriminator between PD and controls, it remained in the regression analyses as it is a widely used and validated tool for detection of MCI.

**Table 3:** Regression SDMT raw scores, SDMT corrected scores, and MoCA scores.

Model **SDMT** **Raw Scores**	Unstandardized β	Standard Error	Standardized β	t	p	95 %CI
Intercept	82.04	9.68		8.47	<0.001	62.8, 101.3
Age	−0.63	0.13	−0.43	−4.99	<0.001	−0.89, −0.38
Diagnosis	−10.13	2.18	−0.40	−4.66	<0.001	−14.45, −5.81
Education	0.48	0.40	0.10	1.20	0.23	−0.31, 1.26

Model **SDMT** **T-Scores**						
Intercept	67.34	9.40		7.18	<0.001	48.71, 86.00
Age	−0.23	0.12	−0.18	−1.88	0.63	−0.47, 0.01
Diagnosis	−8.44	2.11	−0.38	−4.00	<0.001	−12.63, −4.25
Education	−0.40	0.38	−0.10	−1.04	0.30	1.16, 0.37

Model **MoCA**						
Intercept	28.76	3.18		9.05	<0.001	22.45, 35.07
Age	−0.12	0.04	−0.27	−2.79	0.006	−0.20, −0.03
Diagnosis	−1.85	0.71	−0.25	−2.59	0.011	−3.27, −0.43
Education	0.31	0.13	0.23	2.37	0.20	0.05, 0.57

The MoCA domain-specific index scores were assessed to measure if demographic factors or diagnosis were significantly related to scores. Factors associated with each of the sub scores were: 1) diagnosis (β = −0.88, CI [−1.57, −0.20]) for the **Memory** index, 2) age (β = −0.03, CI [-0.05, −0.002]) and diagnosis (β = −0.63, CI [- 1.06, −0.20]) for **Visuospatial/ Executive Function**, 3) age (β = −0.05, CI [−0.07, −0.02]) and education (β = −0.12, CI [ 0.43, 0.21]) for **Attention**, 4) age (β = −0.12, CI [−0.02, −0.003]) for **Naming**, 5) education (β = −0.10, CI [ 0.05, 0.15])for **Abstraction**, while 6) **Language** and **Orientation** did not have any significant factors.

Finally, correlation analysis explored the relationship between SDMT, SDMT T-scores, and MoCA domain specific index scores. Significant correlations were found between SDMT raw scores and MoCA memory (r = 0.41, p < 0.001), visuospatial/executive function (r = 0.50, p < 0.001), attention (r = 0.41, p < 0.001), naming (r = 0.37, p < 0.001), and language (r = 0.26, p = 0.011). Significant correlations were measured between SDMT T-scores and MoCA memory (r = 0.42, p < 0.001), visuospatial/executive function (r = 0.46, p < 0.001), attention (r = 0.26, p = 0.011), and naming (r = 0.29, p = 0.005). (Table 4).

**Table 4:** Correlations.

	SDMT raw score	SDMT T-score
Memory	r = 0.41 p < 0.001	r = 0.42 p < 0.001
Visuospatial/ Executive Functioning	r = 0.50 p < 0.001	r = 0.46 p < 0.001
Attention	r = 0.41 p < 0.001	r = 0.26 p = 0.011
Naming	r = 0.37 p < 0.001	r = 0.29 p = 0.005
Language	r = 0.26 p = 0.011	r = 0.19 p = 0.07
Orientation	r = -0.10 p = 0.568	r = -0.10 p = 0.662

## 4 Discussion

In the realm of neurodegenerative disorders like PD, there is a pressing demand for reliable tools that can effectively screen for cognitive impairment. Both clinicians and researchers need instruments that offer consistent and replicable results, enabling them to accurately track cognitive changes over time. The SDMT emerged as a promising candidate, demonstrating its ability to distinguish individuals with cognitive impairment from those without, and exhibiting strong correlations with established cognitive assessment methods. Our research findings underscored this potential, revealing that participants with PD consistently scored lower than the control group across all three screening tests: MoCA, SDMT, and KD. However, after applying the Bonferroni correction, only the SDMT proved statistically significant in discerning the disparities between the two groups. This highlights the SDMT's effectiveness, making it a useful tool for detecting cognitive impairment in individuals afflicted with PD.

The MoCA has been previously validated for the detection of cognitive impairment not only in PD [5] but also in various other medical conditions such as mild cognitive impairment and mild Alzheimer’s disease [18]. In our study, we observed noteworthy correlations between poor performance on the SDMT and MoCA memory, visuospatial/executive function, naming, attention, and language. This indicates that the SDMT captured impairment in multiple cognitive domains underscoring the utility of the SDMT beyond attention, memory, and information processing speed.

There is a growing concern that the MoCA's sensitivity might be a double-edged sword. While its sensitivity allows for the detection of subtle cognitive changes, it may also lead to a higher rate of false positives, potentially causing unnecessary distress and further diagnostic testing for individuals without cognitive impairment [19]. This concern is particularly relevant in diverse groups where factors such as age, education, and cultural background may influence MoCA scores [20,21]. In our study, the MoCA categorized 73 % of participants as impaired, while the SDMT only placed 60 % of participants in that category. Notably, the SDMT incorporates age and education as crucial factors when assessing cognitive performance, whereas the MoCA only accounts for education. This distinction likely contributed to a higher number of participants being identified as cognitively impaired on the MoCA compared to the SDMT.

We completed analyses exploring which factors were significant predictors of MoCA and SDMT scores with age, education, and diagnosis included in the models. Age, education, and diagnosis were significant factors of the MoCA. Age and diagnosis were factors for SDMT raw scores, but only diagnosis remained once age and education-based norms were applied. These findings suggest that MoCA scores tend to decline with age, but the cutoff for cognitive impairment does not take that into account and can lead to false positives and potential unnecessary distress in older adults. Given that age significantly influences cognitive performance [[22], [23], [24]] the lack of adjustment for age in scoring the MoCA may be inadequate and could lead to an overestimation of cognitive impairment based on our findings. Combined screening using the SDMT and MoCA may improve the sensitivity and specificity of screening for cognitive impairment.

Researchers and clinicians must consider these limitations when using the MoCA as a cognitive screening tool and interpret its results cautiously, considering individual characteristics and the potential for false positives in sensitive populations. This issue underscores the ongoing need for the development of more specific and culturally unbiased cognitive assessment tools that can complement the MoCA in clinical practice.

The SDMT is a well-established standardized neuropsychological assessment tool commonly used in patients with MS [25]. However, research studies have consistently demonstrated its effectiveness in detecting cognitive deficits across a range of neurological conditions, including but not limited to PD [26]. The SDMT's ability to assess processing speed, attention, and working memory makes it particularly well suited to identify cognitive changes in neurodegenerative disorders where these cognitive domains are often compromised [9,27]. The ability to use an oral version is useful in disorders that have motor dysfunction such as PD. Pascoe et.al. found diminished SDMT scores in participants with PD and preserved oculomotor function showing that slowed motor function did not impact scores [9].

Norms for the SDMT are crucial for interpreting individual test scores and were established using a large and diverse sample of participants. Smith et al. [16] used a large sample of healthy individuals to establish baseline performance levels, allowing clinicians and researchers to compare an individual's SDMT score to the normative data and assess their cognitive functioning. These normative data help clinicians screen for cognitive impairment, track changes over time, and inform treatment strategies in various clinical settings.

We conducted an investigation into the KD test's potential validity as a screening tool for cognitive impairment in PD. The KD test, originally developed as an assessment tool for reading proficiency,[10] gained attention for its potential utility in detecting cognitive deficits across various clinical groups. However, when we compared the KD test to established cognitive screening measures such as the MoCA and the SDMT, our cohort yielded no significant results. Despite its intriguing history and initial promise, the KD test failed to demonstrate superiority or even comparability to these well-established assessments. The KD may not assess the cognitive domains associated with PD as well as it may in other neurological conditions such as acute concussion.

Age is a critical factor that significantly impacts cognitive performance with gradual decline as individuals age. One of the most notable effects of aging on cognition is a decrease in processing speed [22,23]. Slower processing speed can affect various cognitive functions, such as memory, attention, and problem-solving. Additionally, older adults often experience challenges in working memory, which can influence tasks requiring complex information processing [24]. Furthermore, age-related cognitive changes can also be influenced by factors such as education, genetics, and lifestyle and higher levels of education have been associated with better cognitive performance in older age [28]. Understanding the intricate interplay of these factors and their impact on cognitive performance in aging individuals is crucial for developing effective strategies to promote and assess cognitive health.

In conclusion, our findings bolster the research supporting the utility of the SDMT as a cognitive screening tool in PD. The value of the SDMT lies in its comprehensive approach to normative data, which considers both age and education. Our results not only demonstrate that the SDMT is comparable to the MoCA but also suggest its potential superiority in identifying cognitive impairment within this specific patient sample. By considering both age and education, the SDMT provides a more precise and holistic evaluation of cognitive function, surpassing the MoCA's reliance solely on educational attainment as a correction factor. This highlights the SDMT as a valuable tool for clinicians and researchers in the early detection and monitoring of cognitive deficits in people with PD.

The major limitations of this study include a small sample size, the lack of a comprehensive neuropsychological test battery as a gold standard for diagnosing cognitive impairment, and the absence of consideration for certain demographic features such as race, socioeconomic status, and gender, which could potentially influence cognitive performance. For example, one study found that African American individuals with PD exhibited a higher prevalence of mild cognitive impairment compared to their Caucasian counterparts [29]. This disparity may be attributed to individual variations, differences in access to healthcare, and socioeconomic factors. Gender, too, plays a role in cognitive outcomes. Reekes, et. al. found that men with PD had poorer performance on executive function and processing speed compared to women [30]. Recognizing the interplay of race and gender in the cognitive performance of people with PD is vital for developing more precise diagnostic and treatment strategies tailored to everyone’s unique needs.

Future research endeavors will explore the utility of the SDMT as a cognitive screening tool within a broader spectrum of neurological disorders where its validation is currently lacking. This prospective research direction is poised to enhance our understanding of the SDMT's applicability beyond its existing validated domains, thereby contributing to the refinement of diagnostic and therapeutic strategies in neurological disorders.

## Ethics statement

This study was reviewed and approved by the LSUHSC – New Orleans Institutional Review Board with reference #1104. All participants completed the informed consent process. No patient identifiable information is included in the manuscript.

## CRediT authorship contribution statement

**Daniyell Thomason:** Writing – review & editing, Writing – original draft, Formal analysis, Data curation. **Morganne Manuel:** Writing – review & editing, Methodology, Formal analysis, Data curation, Conceptualization. **Shannin Moody:** Writing – review & editing, Methodology, Investigation, Formal analysis, Data curation. **Jesus Lovera:** Writing – review & editing, Methodology, Investigation, Data curation, Conceptualization. **Brain Copeland:** Writing – review & editing, Methodology, Investigation, Data curation. **Deidre Devier:** Writing – review & editing, Supervision, Project administration, Methodology, Investigation, Formal analysis, Data curation, Conceptualization.

## Declaration of competing interest

The authors declare that they have no known competing financial interests or personal relationships that could have appeared to influence the work reported in this paper.
